# Immune checkpoint inhibitors in cancer therapy: what lies beyond monoclonal antibodies?

**DOI:** 10.1007/s12032-025-02822-1

**Published:** 2025-06-19

**Authors:** Mohammad Reza Zamani, Pavel Šácha

**Affiliations:** 1 Department of Cell Biology, Faculty of Science, Charles University, Prague, Czech Republic; 2 Institute of Organic Chemistry and Biochemistry, Czech Academy of Sciences, Flemingovo n. 2, Prague 6, Prague, 16610 Czech Republic

**Keywords:** Cancer therapy, Immunotherapy, Immune checkpoint inhibitors, Monoclonal antibodies, Small molecules

## Abstract

Immune checkpoints are critical in modulating immune responses and maintaining self-tolerance. Cancer cells can exploit these mechanisms to evade immune detection, making immune checkpoints attractive targets for cancer therapy. The introduction of immune checkpoint inhibitors (ICIs) has transformed cancer treatment, with monoclonal antibodies targeting CTLA-4, PD-1, and PD-L1 demonstrating clinical success. However, challenges such as immune-related adverse events, primary and acquired resistance, and high treatment costs persist. To address these challenges, it is essential to explore alternative strategies, including small-molecule and peptide-based inhibitors, aptamers, RNA-based therapies, gene-editing technologies, bispecific and multispecific agents, and cell-based therapies. Additionally, innovative approaches such as lysosome-targeting chimeras, proteolysis-targeting chimeras, and *N*-(2-hydroxypropyl) methacrylamide copolymers are emerging as promising options for enhancing treatment effectiveness. This review highlights significant advancements in the field, focusing on their clinical implications and successes.

## Introduction

Inhibitory immune checkpoints play a crucial role in maintaining immune homeostasis by regulating the intensity and duration of immune responses. These checkpoints primarily comprise surface receptors interacting with specific ligands to dampen immune activation and prevent excessive tissue damage. This regulatory mechanism is essential for preventing autoimmune reactions, while preserving tolerance to self-antigens. However, cancer cells can evade immune surveillance by exploiting these checkpoints, which are prime targets for cancer immunotherapy [1].

Monoclonal antibodies specifically targeting immune checkpoints such as Cytotoxic T-Lymphocyte-Associated Protein 4 (CTLA-4) and Programmed Cell Death Protein 1 (PD-1) on T cells can effectively modulate immune responses against tumors. Designed to bind to specific antigens, these biologics disrupt the inhibitory pathways that cancer cells use to evade immune surveillance, enhancing the immune system’s ability to eliminate malignant cells [2]. Food and Drug Administration (FDA)-approved monoclonal antibodies have demonstrated remarkable efficacy across various malignancies, significantly improving patient outcomes [3].

Although monoclonal antibody-based immune checkpoint inhibitors (ICIs) are effective, they come with notable drawbacks. Immune-related adverse events (irAEs), which stem from systemic immune activation, can affect multiple organ systems and range from mild to severe. Additionally, not all patients respond to ICIs, with some experiencing primary or acquired resistance over time[4]. The high cost of these biological therapies adds a substantial economic burden, limiting their accessibility for many patients.

To address these limitations and expand the therapeutic arsenal, researchers are investigating various alternative approaches to immune checkpoint modulation. These include small-molecule and peptide-based inhibitors, aptamers, gene-editing technologies (e.g., CRISPR), and RNA-based therapies (e.g., siRNA). Additionally, innovative strategies such as bispecific and multispecific agents, lysosome-targeting chimeras (LYTACs), proteolysis-targeting chimeras (PROTACs), and N-(2-hydroxypropyl) methacrylamide (HPMA) copolymer-based drug delivery systems are advancing as promising avenues for therapeutic development.

Beyond these molecular strategies, cell- and virus-based platforms, such as CAR-NK cells and oncolytic viruses, have emerged as novel tools for checkpoint modulation. By combining direct tumor killing with immune microenvironment remodeling, they offer unique synergy with checkpoint inhibitors and serve as promising complements or alternatives to monoclonal antibodies [5, 6].

**Fig. 1:**
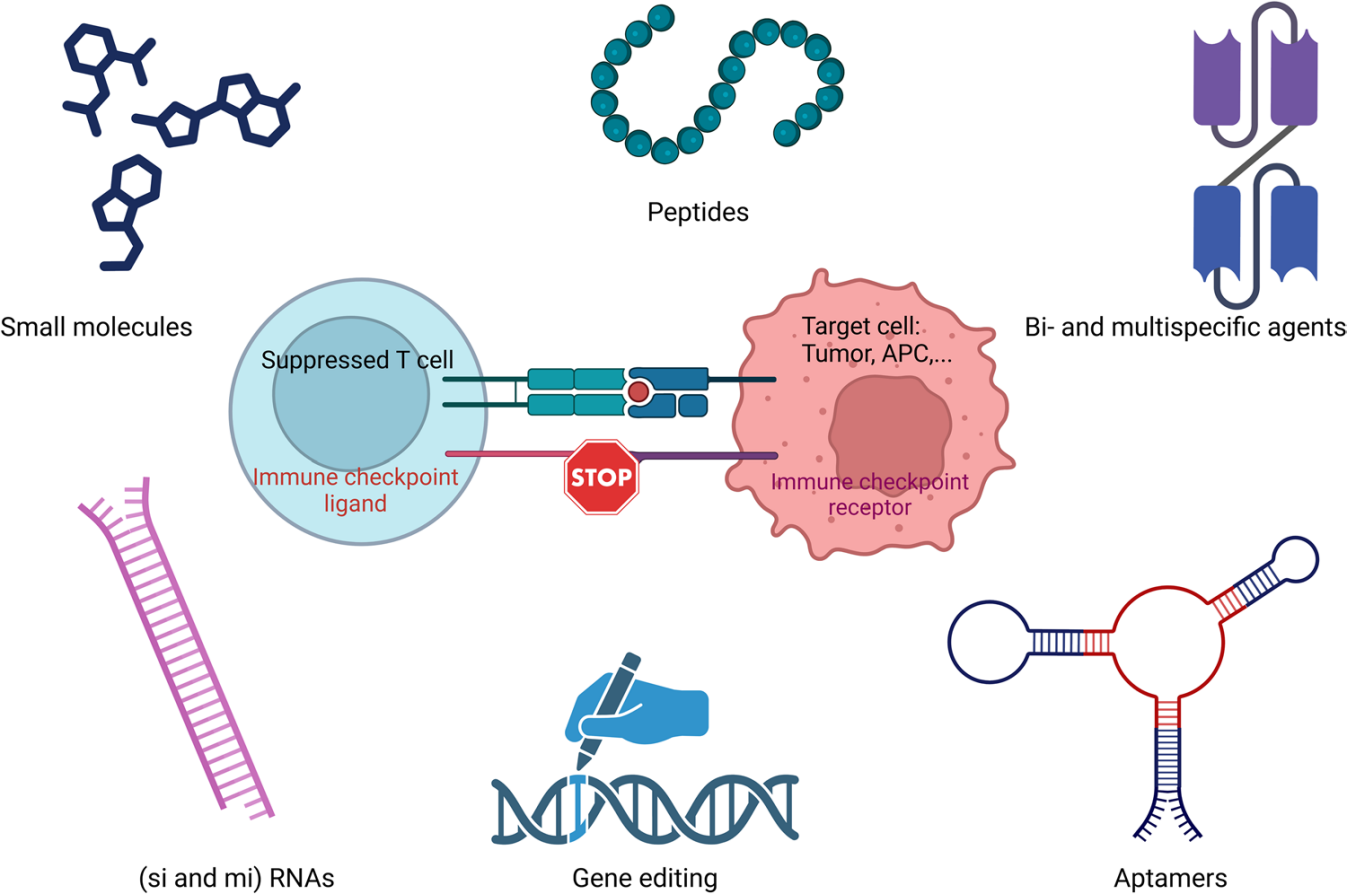
Alternatives to monoclonal antibodies for targeting inhibitory immune checkpoints in cancer immunotherapy (Created with bioRender.com)

This review highlights the latest advances and remaining challenges in cancer immunotherapy, focusing on notable alternative approaches to traditional monoclonal antibodies for targeting inhibitory immune checkpoints (Fig. 1).

## Inhibitory immune checkpoints

CTLA-4 and PD-1, along with their ligands CD80/CD86 and PD-L1/PD-L2, have attracted considerable research interest due to their roles in immune regulation and their potential for therapeutic applications [7]. Indeed, the advent of ICIs targeting CTLA-4, PD-1, and PD-L1 transformed cancer therapy [2]. Other inhibitory checkpoints such as Lymphocyte Activation Gene 3 (LAG-3), T Cell Immunoglobulin and Mucin-3 (TIM-3), T Cell Immunoreceptor with Ig and ITIM domains (TIGIT), and V-domain Ig Suppressor of T Cell Activation (VISTA) are being actively studied to determine their roles in immune modulation and potential as therapeutic targets [8].

**Fig. 2:**
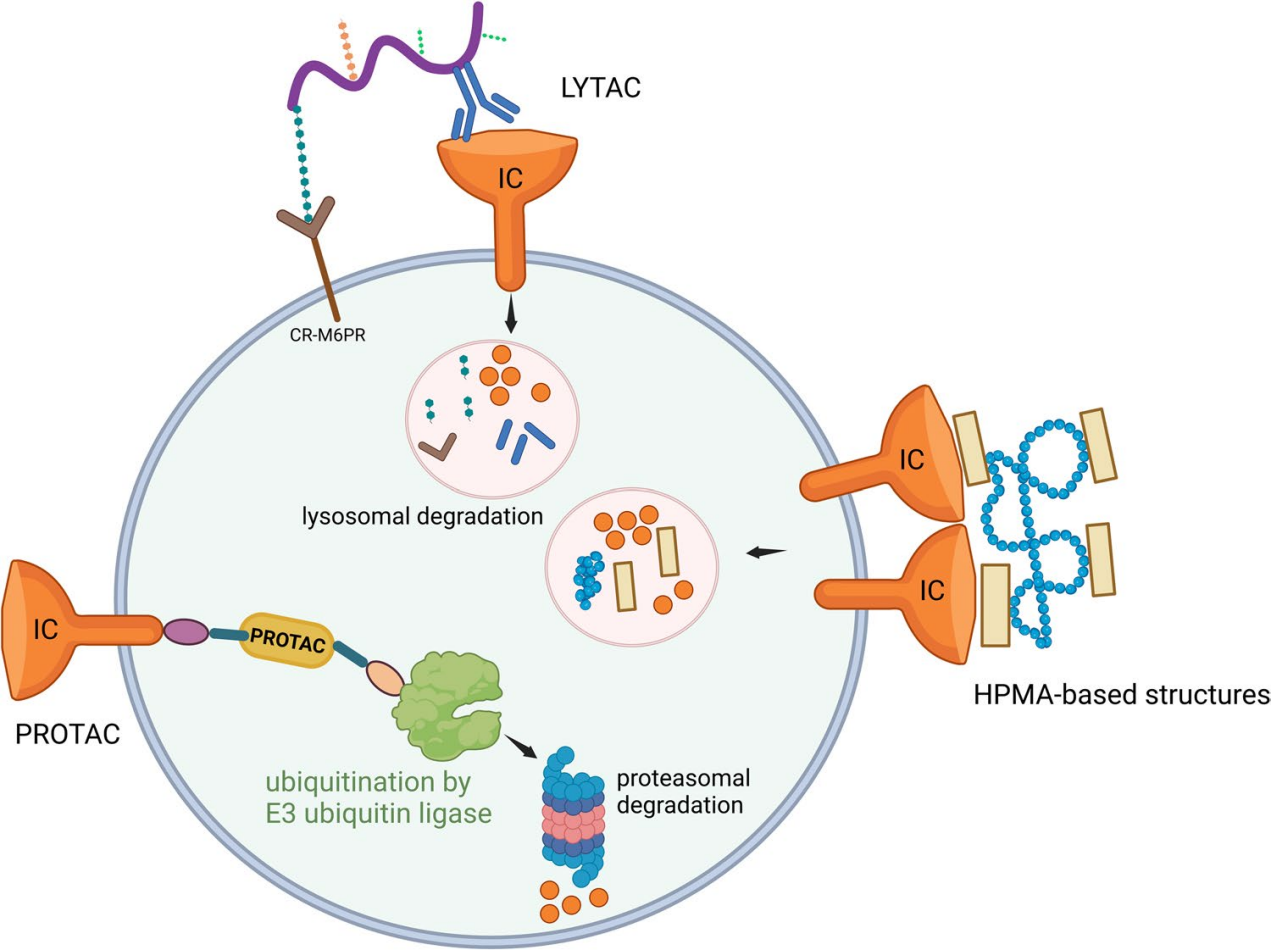
Emerging approaches for targeting and degrading membrane immune checkpoints (ICs). The mechanisms of action are not fully understood. Cation-independent mannose 6-phosphate receptor (CR-M6PR). (Created with bioRender.com)

The mechanism of action of ICIs, particularly monoclonal antibodies, focuses on blocking the protein–protein interactions that tumors exploit to evade immune responses (Table 1). Strategies also include promoting protein dimerization. In addition to preventing receptor–ligand interactions, dimerization can induce protein internalization and directly reduce the concentration of the target on the cell surface (Fig. 2), decreasing the target’s availability for immune evasion [9]. Researchers are developing strategies to harness immune checkpoint internalization for drug delivery, while also working to prevent receptor degradation to sustain immune activation and enhance therapeutic outcomes.

## Therapeutic monoclonal antibodies

Monoclonal antibodies have become central to modern cancer immunotherapy by targeting immune checkpoints and restoring immune surveillance against tumors [10]. These biologics disrupt inhibitory pathways exploited by cancer cells, enabling the immune system to eliminate malignant cells. FDA approval of the ICIs ipilimumab, pembrolizumab, nivolumab, atezolizumab, durvalumab, and avelumab underscores the success of monoclonal antibodies at harnessing the body's own defenses against cancer [2].

Ipilimumab, the first FDA-approved CTLA-4 inhibitor, has demonstrated efficacy for treatment of advanced melanoma and as part of combination therapies for renal cell carcinoma and colorectal cancer [11]. Pembrolizumab and nivolumab, target PD-1, have improved patient survival rates in patients with melanoma, non-small cell lung cancer (NSCLC), and Hodgkin lymphoma. Additionally, PD-L1 inhibitors atezolizumab, durvalumab, and avelumab have been successful for treating urothelial carcinoma, NSCLC, and Merkel cell carcinoma [12]. Ongoing research is exploring novel immune checkpoints, such as LAG-3, TIM-3, and TIGIT, as potential targets for future antibody-based interventions [13].

One unique and natural characteristic of the antibodies is the Fc region, which may enhance immune activation through mechanisms such as antibody-dependent cellular cytotoxicity (ADCC), antibody-dependent cellular phagocytosis (ADCP), and complement activation, prolong antibody half-life via FcRn recycling, and modulate immune cell activity, improving antitumor responses. For example, Ipilimumab has a functional Fc region, allowing for immune cell engagement [14]. However, Fc functionality may also increase the risk of irAEs and thus contribute to antibody-dependent enhancement by depleting regulatory T cells, leading to autoimmunity, and potentially limiting tumor penetration [15]. Therefore, antibodies such as nivolumab and pembrolizumab, which have modified IgG4 Fc regions, are engineered to reduce Fc receptor interactions, minimizing off-target effects and toxicity [16].

Despite the efficacy of clinical immune checkpoint antibodies, these treatments have limitations. irAEs can range from mild to severe, affecting a variety of organ systems due to the systemic immune activation induced by these therapies [17]. Furthermore, some patients have a low response rate to ICIs. Additionally, the high cost of these biologics poses a significant economic burden, restricting their accessibility [18].

The lack of response to ICIs experienced by some patients is mainly due to either primary or acquired resistance mechanisms. Certain cancers, such as pancreatic and prostate cancers, often exhibit primary resistance to ICIs [19]. Primary resistance can occur when tumors have low mutational burdens, lack PD-L1 expression, or create an immunosuppressive microenvironment that excludes T cells. On the other hand, cancers like melanoma and NSCLC can initially respond to ICIs but later develop acquired resistance [20]. This can occur when tumors adapt by upregulating alternative checkpoints, losing antigen presentation capabilities, or causing T cell exhaustion [21]. These challenges highlight the complexity of ICI resistance and the need for continued research and combination therapies to improve patient outcomes (Fig. 1).

## Small-molecule inhibitors

Small-molecule ICIs, those with molecular weights below 1,000 g/mol, offer several advantages compared to monoclonal antibodies. These include oral bioavailability, easier synthesis, and lower costs associated with manufacturing, storage, and transportation. Additionally, these drugs have improved tissue permeability and penetration, a reduced risk of immunogenicity, and the potential to minimize side effects and off-target toxicities through optimized pharmacokinetics (Table 1) [22].

Designing small-molecule inhibitors is difficult due to the complex structures of immune checkpoint proteins like PD-1/PD-L1 and CTLA-4, which require precise binding interactions. Their small size makes it challenging to effectively inhibit large protein–protein interactions, as peptides typically cover a contact area of 1500–3000 square angstroms (Å^2^), while small molecules cover only 300–1000 Å^2^ of the protein surface. Additionally, the typically shorter half-life of small molecules requires formulation improvements or chemical modifications to enhance stability and reduce metabolic degradation [23].

Strategies to address these challenges include structure-based design, medicinal chemistry optimization, prodrug strategies, nanoparticle delivery systems, and combination therapies. Extensive toxicity screening and biomarker monitoring are crucial for managing adverse effects and refining treatment approaches [23].

CA-170, an oral small-molecule dual inhibitor targeting the immune checkpoints PD-L1 and VISTA, has demonstrated promising results across various clinical trials. Initially, there were doubts about its efficacy on the checkpoint itself, but later research revealed that CA-170 forms a ternary complex with PD-L1, blocking inhibitory signal transduction and potentially activating T cells [24].

In the first Phase 1 clinical evaluation, CA-170 showed a favorable safety profile in patients with advanced solid tumors or lymphomas, with some patients experiencing tumor regression, and was well tolerated at doses up to 1200 mg. Notably, 8 patients experienced tumor regression from baseline, indicating preliminary antitumor activity [25]. It is currently being studied in the clinical trial registered as CTRI/2021/10/037699, titled “A Phase 2b/3 clinical trial to assess the safety and efficacy of study drug, CA-170 in combination with standard chemotherapy in stage IV lung cancer patients.” The trial is open to recruitment [26].

Further diversifying the landscape of small-molecule inhibitors, YPD-30 (IMMH-010) emerges as another addition. YPD-30 promotes PD-L1 dimerization and internalization, thereby downregulating PD-L1 levels and subsequently activating T lymphocytes [27]. After oral administration, it had significant antitumor activity and favorable pharmacokinetic characteristics. Due to its notable safety and tolerability in both single and multiple doses, YPD-30 has advanced to Phase 1 clinical trials in China [28].

The clinical trial NCT04122339 is a Phase 1 study assessing the orally active small PD-1/PD-L1 inhibitor MAX-10181 in patients with advanced solid tumors, conducted in Australia. The primary outcomes include adverse events, maximum tolerated dose, and the recommended phase dose [29]. It is reportedly being evaluated in combination with capecitabine in patients with advanced or metastatic solid tumors in Phase 2 (CTR20242270) [30]. One notable characteristic of this molecule is its ability to cross the blood–brain barrier, as demonstrated in a GL-261 animal model study, where the combination of MAX-10181 with temozolomide significantly extended survival by 50% compared to temozolomide monotherapy [31].

Similarly, GS-4224 is an orally bioavailable small molecule that targets PD-L1, causing it to dimerize and block its interaction with PD-1. In Phase 1b/2 study with advanced solid tumor patients (clinical trial NCT04049617), GS-4224 was well tolerated at doses ranging from 400 to 1500 mg daily [32]. The treatment increased plasma levels of GS-4224 reduced free PD-L1 on T cells, and elevated plasma cytokines and chemokines [33]. The trial was ultimately terminated due to sponsor decision [32].

Bristol-Myers Squibb is known for its biologic therapies in immune checkpoint inhibition, but its small-molecule PD-L1 inhibitors, BMS-200 and BMS-202, have not yet reached the same clinical prominence. These small molecules bind to PD-L1, preventing its interaction with PD-1 and disrupting the immune checkpoint pathway. BMS-202 (IC50 of 18 nM) has shown promising results in preclinical studies, such as reducing tumor size in mouse melanoma models [34]. Another example is ARB-272572, which demonstrated highly potent activity with a 400 pM IC50 in cell-free PD-1/PD-L1 HTRF assays [35], an uncommon result for small molecules targeting protein–protein interactions.

## Peptide-based inhibitors

Peptide-based ICIs contrast with small-molecule inhibitors in terms of size, specificity, and delivery methods. For example, peptides designed to disrupt the PD-1/PD-L1 pathway mimic the binding sites of PD-1 or PD-L1, effectively blocking their interaction and thereby enhancing T cell activity against cancer cells (Table 1). Peptide-based inhibitors are also being investigated for their potential against checkpoints of emerging interest including LAG-3, TIM-3, and TIGIT [36].

Despite their advantages over small molecules and large biologics in certain areas, peptides face significant challenges with stability, specificity, and half-life. Peptides composed of L-amino acids often exhibit suboptimal stability, largely due to rapid degradation by proteolytic enzymes, leading to difficulties in achieving robust target blockade. While peptide cyclization can improve conformational stability and reduce enzymatic degradation, peptides still struggle to match the high specificity and strong binding of antibodies, which benefit from their complex structure. Additionally, like small molecules, peptides typically have relatively short half-lives and rapid clearance rates, complicating their therapeutic use [37]. These limitations have slowed the advancement of peptide-based inhibitors.

However, at least one peptide-based inhibitor has advanced to human clinical trials. BMS-986189, a macrocyclic peptide developed by Bristol-Myers Squibb (BMS) to target PD-L1, was well tolerated with manageable side effects among phase 1 clinical trial participants with advanced solid tumors (NCT02739373). Pharmacokinetic analysis indicated that the drug had predictable and dose-linear behavior. Preliminary efficacy results showed promise, with indications of potential antitumor activity. These findings support the continued development of BMS-986189, although further clinical trials are needed to assess its efficacy in a wider variety of tumors and patient groups [38].

## Aptamers

Aptamers are single-stranded RNA or DNA, typically less than 100 nucleotides long, that exhibit high affinity and specificity for their target molecules, earning them the nickname ‘chemical antibodies’ [39]. Identified through the systematic evolution of ligands by exponential enrichment (SELEX), their high structural flexibility allows them to form intricate binding clefts. This flexibility theoretically enables more precise interactions with targets compared to antibodies [40]. Similar to antibodies, aptamers can selectively bind to specific proteins, including immune checkpoint receptors or their ligands, disrupting their interaction and inhibiting suppressive signals that dampen immune responses (Table 1) [41, 42].

Despite their promising attributes, aptamers have some properties that limit their clinical utility. These include vulnerability of unmodified aptamers to nuclease degradation, difficulties in efficient delivery to target tissues, and relatively short half-lives [43]. Chemical modifications can improve aptamer stability and protect against nuclease degradation, while sophisticated delivery systems such as nanoparticles can enhance targeting efficiency. Careful design and screening are also crucial to achieve high affinity, specific binding with minimal off-target effects.

As an example, a bispecific aptamer (BiApt) targeting PD-1 and nucleolin, a protein which is highly expressed on the surface of multiple cancer cells, demonstrated promising results in a murine colon cancer model, effectively inhibiting tumor growth and enhancing T cell recruitment to the tumor site, boosting antitumor immunity (Table 1) [44]. Similarly, a PDGFRβ-targeting aptamer demonstrated synergistic effects when combined with immune checkpoint blockade in a syngeneic mouse model of triple-negative breast cancer. This aptamer specifically bound to PDGFRβ-positive cells, inhibited cell migration, and reduced tumor growth when used alongside anti-PD-L1 antibodies [45].

Moreover, growing in vivo studies mainly targeting PD-1/PD-L1 and CTLA-4 highlight the potential of using aptamers in the future of immunotherapy [42, 46, 47].

## RNA-based therapies

Small interfering RNA (siRNA) and microRNA (miRNA) have emerged as innovative tools to target immune checkpoints. These RNA molecules modulate gene expression post-transcriptionally, potentially exerting significant influence on the immune response [48, 49].

RNA-based cancer therapies face considerable challenges compared to monoclonal antibodies [50]. Delivery is a major hurdle, as advanced systems are required to ensure intact RNA molecules reach target cells, unlike the direct and precise targeting of antibodies. RNA therapies may induce off-target effects by interacting with other cellular pathways, whereas antibodies typically exhibit more specific binding. Additionally, RNA molecules can stimulate immune responses, potentially causing adverse effects not commonly seen with antibodies. The regulatory approval process for RNA therapies is stringent due to safety and efficacy concerns, given their innovative nature [51].

Several miRNAs have demonstrated efficacy as ICIs in vivo through targeting key pathways. miR-138 has been shown to target the PD-1/PD-L1 pathway by downregulating PD-1 expression on T cells or PD-L1 expression on tumor cells in various cancer models, thereby enhancing T cell-mediated immune responses against tumors [52, 53]. miR-34a also acts on the PD-1/PD-L1 pathway by targeting PD-L1 mRNA in cancer cells, leading to decreased PD-L1 expression and increased cytotoxic T cell activity [54, 55]. miR-200 regulates the PD-L1 pathway by decreasing PD-L1 levels in cancer cells, potentially alleviating immune suppression and enhancing immune-mediated tumor clearance [56]. Additionally, miR-424 targets the CTLA-4 pathway by reducing CTLA-4 expression in T cells, thereby enhancing T cell activation and proliferation, which can potentiate antitumor immune responses in vivo [57].

While these miRNAs effectively target pathways such as PD-1/PD-L1 and CTLA-4 in preclinical models, achieving precise targeting of tumor cells in humans without affecting healthy tissues remains a challenge.

## Gene-editing techniques

Gene-editing techniques involve precise modification of genes associated with immune checkpoint pathways in both cancer cells and immune cells. By employing advanced techniques like CRISPR-Cas9, researchers can specifically target and disrupt the expression of immune checkpoint proteins, such as PD-1/PD-L1 and CTLA-4. This targeted disruption can potentially improve the immune system’s ability to overcome the suppressive signals these proteins create, thereby boosting the overall efficacy of cancer immunotherapy [58, 59].

Despite the promise of gene-editing technologies, determining their safety remains paramount, with long-term monitoring essential to detect adverse effects and ensure the durability of therapeutic responses [60]. Among these challenges are mutation rates, mosaicism, and off-target effects in gene editing, which collectively reflect the frequency of intended (on-target) or unintended (off-target) mutations, as well as the technique’s efficiency, precision, and safety. Optimizing these factors is crucial for developing reliable gene-editing technologies [61].

The clinical trial NCT02793856 assessed the safety and feasibility of using CRISPR-Cas9-edited PD-1 knockout T cells in patients with advanced NSCLC. The study included 22 patients, 17 of whom had sufficiently edited T cells for infusion. All treatment-related adverse events were classified as grade 1 or 2, indicating good tolerability. Post-infusion, edited T cells were detectable in the peripheral blood of the patients. The median progression-free survival (PFS) was 7.7 weeks, and the median overall survival was 42.6 weeks. Off-target mutations were rare, with a median frequency of 0.05% at 18 candidate sites. These results suggest that CRISPR-Cas9-edited T cell therapy is generally safe and feasible for treating advanced NSCLC. However, while the trial confirmed the feasibility of this approach, it highlighted the need for further research to evaluate its efficacy and optimize the treatment protocols [62, 63].

A pilot dose escalation study (NCT03747965) examining mesothelin-directed CAR-T cells with PD-1 disruption (GC008t) in patients with advanced solid tumors that express mesothelin, confirmed that using CRISPR to inactivate PD-1 in CAR-T cells is both feasible and safe. Among the seven evaluable patients, the best responses observed were stable disease in four patients and partial response in two patients (dosed at ≥ 1 × 10⁷ units/kg), with PFS of 80 and 160 days, respectively. However, the genetic modification did not lead to significant improvements in CAR-T cell expansion and persistence, despite the presence of natural TCR and the use of lymphodepletion [64].

The Phase 1 trial of CRISPR-modified MPTK-CAR-T cells targeting mesothelin-positive solid tumors also demonstrated safety, with no severe toxicities and manageable side effects, and feasibility in producing and delivering the cells. Unlike the previously mentioned NCT03747965 trial, this study focuses on dual gene disruptions—both PD-1 and the T cell receptor (TCR). Preliminary antitumor activity was observed, with some patients showing stable disease or partial responses, though no complete responses were achieved. The disruption of PD-1 reduced immune suppression, while TCR deletion minimized the risk of graft-versus-host disease. These findings suggest the therapy is safe with potential efficacy, supporting further investigation.

Taking the results of these two examples together, TCR knockout could emphasize safety and wider applicability, whereas PD-1 knockout alone may result in greater immediate efficacy for certain patients.

These trials also highlight efficacy limitations, demonstrated by modest disease control rates and relatively short PFS. Unlike mAbs, which are well-established therapies offering predictable short- to medium-term PFS benefits, gene-editing techniques such as CRISPR are experimental but highly precise, with the potential to achieve durable or even curative PFS outcomes, particularly in engineered immune therapies like CAR-T.

In general, genetically modified cells often show inconsistent expansion and persistence in patients, which are critical factors for achieving durable therapeutic effects. Solutions may involve refining gene-editing methods to improve CAR-T cell durability and functionality, which involves precise modifications to enhance CAR-T cell survival, resistance to exhaustion, and ability to thrive in the tumor microenvironment. This includes targeting genes that boost cell persistence, using accurate editing tools like CRISPR to reduce off-target effects, and promoting memory-like CAR-T cells for long-term efficacy. Additionally, edits can increase resistance to suppressive signals from tumors, while optimized manufacturing techniques help create robust, highly functional CAR-T cells.

## Bispecific and multispecific agents

Bispecific and multispecific agents are at the forefront of alternative immune checkpoint therapy, offering a sophisticated approach to enhance cancer treatment precision and efficacy. These agents are engineered to concurrently target multiple antigens or immune checkpoints, thereby maximizing the immune response against tumors. By binding to two or more distinct targets, they bring together different components of the immune system to effectively attack cancer cells, surpassing the capabilities of monospecific agents (Table 1) [65].

**Table 1:** Selected immune checkpoint inhibitors, their structures, target molecules, and the most recent stage of testing

Substance	Structure	Target	Most recent tested stage	Disease/model
CA-170		PD-1 and VISTA	Phase 2b/3 CTRI/2021/10/037699	Stage-IV lung cancer patients
YPD-30 (IMMH-010)		PD-L1	Phase 1 NCT04343859	NSCLC
MAX-10181		PD-L1	Phase 2 CTR20242270	Advanced or metastatic solid tumors
GS-4224		PD-L1	Phase 1 NCT04049617	Advanced solid tumors
BMS-202		PD-L1	Preclinical	Murine melanoma
BMS-986189		PD-L1	Phase 1 NCT02739373	Healthy participants
BiApt	DNA nanostructure	PD-1 and nucleolin	Preclinical	Murine colon cancer
XmAb20717 (vudalimab)		CTLA-4 and PD-1	Phase 2 NCT05297903	Advanced biliary tract cancers
lomvastomig (RO7121661)		TIM-3 and PD-1	Phase 2 NCT04785820	Advanced or metastatic squamous-cell carcinoma of the esophagus
ABL501		LAG-3 and PD-L1	Phase 1 NCT05101109	Metastatic solid tumors
Cadonilimab (AK104)		CTLA-4 and PD-1	Phase 3 NCT05008783	Advanced or metastatic gastric or gastroesophageal junction adenocarcinoma

*Note* The drawn antibody structures are schematic representations and only approximate published resources

The development and manufacturing of these agents are complex, leading to high costs and long production times. These agents also pose a higher risk of toxicity due to simultaneous targeting of multiple antigens, which can lead to overactivation of the immune system and damage to healthy tissues [66]. Additionally, their pharmacokinetics may be unpredictable, affecting their stability and distribution in the body. Immunogenicity is another concern, as the body may recognize these complex agents as foreign, reducing their effectiveness [67]. Finally, their novel and intricate mechanisms pose unique regulatory challenges.

One notable Phase 2 trial, NCT05297903, is investigating XmAb20717 (vudalimab), a bispecific antibody targeting both PD-1 and CTLA-4 immune checkpoints. This study aims to evaluate the efficacy and safety of XmAb20717 in patients with advanced solid tumors that are resistant to standard therapies. Preliminary findings from this ongoing study are anticipated to provide insights into XmAb20717’s potential as a treatment option by enhancing immune response against cancer cells [68]. In Phase 1 trial, NCT03517488, 110 patients with advanced solid tumors who had progressed after standard treatments received XmAb20717 at 10 mg/kg every two weeks. The study reported a 13% objective response rate (ORR), including one complete response in melanoma and partial responses in several cancers.

The completed clinical trial NCT03708328 studied lomvastomig (RO7121661), a bispecific antibody targeting PD-1 and TIM-3, in patients with advanced solid tumors like metastatic NSCLC. The trial showed that lomvastomig had an acceptable safety profile and demonstrated clinical activity, especially in patients who had previously received checkpoint inhibitors. Responses were particularly notable in patients with higher PD-L1 expression, suggesting the potential for biomarker-driven treatment strategies. Overall, the results are promising and warrant further investigation [69]. NCT04785820 is a global, multicenter Phase 2 trial designed to evaluate the safety and efficacy of two investigational bispecific antibodies, lomvastomig (RO7121661) and tobemstomig (RO7247669), compared with nivolumab in patients with advanced or metastatic esophageal squamous-cell carcinoma (ESCC) that is resistant or intolerant to prior chemotherapy. The primary endpoint is overall survival, with secondary endpoints including safety, pharmacokinetics, ORR response rate, PFS, and patient-reported outcomes. Although the study is still active, it is no longer recruiting participants [70].

The clinical trial NCT05101109 is evaluating ABL501, a bispecific antibody that targets LAG-3 and PD-L1, in patients with advanced solid tumors. This Phase 1 trial is ongoing, focusing on safety, tolerability, and optimal dosage of the drug. Early findings indicate that ABL501 enhances the activation of immune cells, specifically CD4^+^ and CD8^+^ T cells, which may improve responses in patients with treatment-resistant tumors. The trial will also measure immunogenicity and pharmacokinetics, with final results expected by the end of 2024 [71]. The in vivo results had shown that the bispecific LAG-3xPD-L1 antibody reprogrammed the tumor microenvironment by enhancing dendritic cell activation, promoting antigen cross-presentation, and inducing stronger CD8^+^ T cell activation compared to a combination of anti-LAG-3 and anti-PD-L1 antibodies. This led to improved T cell infiltration, robust antitumor immunity, and significant tumor growth suppression in mouse models, highlighting its therapeutic potential in cancer immunotherapy [72].

Cadonilimab (AK104) is a tetravalent bispecific antibody targeting PD-1 and CTLA-4, while lacking an Fc region. This design prevents ADCC, ADCP, and CDC effects reducing pro-inflammatory cytokine secretion, such as IL-6 and IL-8. These properties may contribute to its lower toxicity profile and enhanced antitumor activity [73]. Early clinical trials had shown promising results in various advanced solid tumors, including cervical cancer, NSCLC, and hepatocellular carcinoma (HCC). These studies have demonstrated cadonilimab’s favorable safety profile and encouraging antitumor activity, particularly in patients with limited treatment options.

Building on these results, the AK104-302 clinical trial is a pivotal Phase 3 study (NCT05008783) evaluating cadonilimab in combination with platinum-based chemotherapy as a first-line treatment for recurrent or metastatic cervical cancer. This trial is significant as it compares the combination treatment against the current standard of care, focusing on overall survival, PFS, and safety. The outcomes of this trial could potentially lead to the approval of cadonilimab as a new treatment option for cervical cancer [73–75].

The NCT03852251 trial investigated the combination of AK104, a bispecific PD-1/CTLA-4 antibody, with modified XELOX chemotherapy in patients with advanced gastric or gastroesophageal junction adenocarcinoma. Results showed that the combination therapy had a manageable safety profile, with an ORR response rate of 60% and a disease control rate of 93.3%. The study demonstrated promising antitumor activity and potential as an effective first-line treatment option for this patient group.

Key limitations of bispecific antibodies identified through clinical trials include variable efficacy across patient groups, inconsistent response rates, and safety concerns, particularly when targeting multiple immune checkpoints. Trials with agents such as XmAb20717 and cadonilimab show potential for dual targeting of PD-1 and CTLA-4, but balancing effective immune activation with toxicity remains important. Similarly, studies with lomvastomig and ABL501 suggest that biomarkers, such as PD-L1 expression, may help predict response, although patient variability complicates this approach. Additional challenges include managing cytokine release syndrome, protecting healthy tissue through precise targeting, and identifying reliable biomarkers to predict treatment outcomes.

Despite the challenges, these trials highlight promising strategies that aim to overcome limitations of traditional therapy, such as resistance and tumor heterogeneity, by leveraging multi-targeted approaches. Potential solutions involve refining patient selection through biomarker analysis to identify responders, optimizing dosing to enhance efficacy and reduce adverse effects, and developing therapies that combine bispecific antibodies with other immunotherapies or chemotherapies to improve outcomes across diverse patient populations.

## Other emerging approaches

Traditional therapies focus on inhibiting immune checkpoints to boost immune responses against tumors. However, arising technologies, such as LYTACs, PROTACs, and HPMA-based drug delivery systems, offer an alternative by promoting the degradation of these immune checkpoint proteins, rather than merely inhibiting them (Fig. 2).

LYTAC (Lysosome-Targeting Chimera) technology is an innovative strategy that targets proteins on the cell surface or outside the cell by marking them for degradation in the lysosome, rather than merely blocking their function like traditional therapies (Fig. 2) [76, 77]. This degradation approach may result in more robust and sustained immune responses, making it more difficult for cancer cells to evade immune detection. While the precise cellular characteristics regulating the behavior of LYTACs in hijacking lysosomal machinery for membrane protein degradation remain largely unknown, a genome-wide screen highlighted the critical role of several endolysosomal genes. Notably, cell surface CI-M6PR receptors were found to be occupied by distinct M6P-modified lysosomal glycoproteins. Disruption of M6P biosynthesis increased the proportion of unoccupied receptors on the cell surface, thereby enhancing LYTAC-receptor engagement and internalization for LYTAC-mediated degradation [78].

However, challenges with this approach remain, including the need for maintaining target specificity to prevent off-target effects and addressing issues with delivery and stability, as the large molecules may degrade before reaching their intended destination. Overcoming these obstacles is crucial to fully unlocking the therapeutic potential of LYTACs in cancer treatment [79].

PROTACs (Proteolysis-Targeting Chimeras) are small, bifunctional molecules that harness the cell’s natural protein degradation system—the ubiquitin–proteasome pathway. One end of the PROTAC binds to the target protein, while the other recruits an E3 ubiquitin ligase. This induced proximity facilitates the transfer of ubiquitin molecules to the target protein, marking it for recognition and breakdown by the proteasome (Fig. 2) [79, 80].

There are several ongoing clinical trials applying this approach to target intracellular signaling pathways [81]. Huntingtin-interacting protein 1-related (HIP1R) plays a key role in targeting immune checkpoints by binding to PD-L1 through its conserved C-terminal domain and using an intrinsic sorting signal to direct PD-L1 to lysosomes for degradation. This process enhances T cell-mediated cytotoxicity, as HIP1R acts as a natural regulator of lysosomal degradation. Moreover, a study has developed the PD-LYSO peptide. This peptide combines the lysosome-sorting signal with the PD-L1-binding sequence of HIP1R, effectively reducing PD-L1 expression in tumor cells [82].

HPMA (*N*-(2-hydroxypropyl)methacrylamide) is a biocompatible, and highly versatile polymer extensively investigated for drug delivery applications (Fig. 2) [83]. In addition to blocking PD-1/PD-L1 interactions, HPMA-based systems have been shown to promote internalization and degradation of PD-L, demonstrating promising antitumor effects both in vitro and in vivo studies [84], with potency comparable to that of monoclonal antibodies [85]. This dual action contributes to the development of polymer-based drug delivery systems that enhance the efficacy of PD-L1 inhibitors.

## Novel platforms based on CAR-NK and oncolytic viruses in checkpoint combination therapy

While this review focuses on alternatives to monoclonal antibodies, emerging cell- and virus-based immunotherapies—particularly chimeric antigen receptor-engineered natural killer (CAR-NK) cells and oncolytic viruses (OVs)—are gaining traction as next-generation platforms to enhance immune checkpoint blockade. These modalities offer distinct immunological mechanisms and synergistic potential that may overcome the limitations of monoclonal antibodies and boost the efficacy of non-antibody-based checkpoint therapies.

### CAR-NK cells

Chimeric antigen receptor natural killer (CAR-NK) cells offer significant advantages over CAR-T cells, including enhanced safety, broader applicability, and synergistic potential with immune checkpoint inhibitors. Unlike CAR-T cells, CAR-NK cells do not require HLA matching, minimizing graft-versus-host disease (GVHD) risks and enabling off-the-shelf use. They exhibit intrinsic antitumor activity through natural cytotoxicity receptors, allowing them to target cancer cells independently of CAR signaling [86]. Additionally, CAR-NK cells have a shorter lifespan, reducing risks of long-term toxicity and cytokine release syndrome (CRS) [87]. Importantly, CAR-NK cells synergize effectively with ICIs, while CAR-T cells often upregulate inhibitory receptors like PD-1 and become exhausted, NK cells maintain robust cytotoxicity and can be further enhanced by checkpoint blockade targeting NK-specific inhibitory receptors (e.g., NKG2A, KIRs) without exacerbating CRS [88]. Their ability to leverage antibody-dependent cellular cytotoxicity (ADCC) alongside monoclonal antibodies further expands their therapeutic versatility. With multiple cell sources (e.g., peripheral blood, cord blood) and scalable production, CAR-NK cells represent a safer, more adaptable platform for next-generation immunotherapy.

Preclinical studies across multiple cancer types demonstrate that combining CAR-NK cell therapy with immune checkpoint inhibitors (ICIs) significantly enhances antitumor efficacy. In castration-resistant prostate cancer, PSMA-targeted CAR-NK-92 cells combined with anti-PD-L1 or anti-PD-1 antibodies overcame IFN-γ-induced PD-L1-mediated resistance, improving tumor control [89]. In glioblastoma, HER2-targeted CAR-NK cells paired with anti-PD-1 therapy not only induced tumor regression and prolonged survival but also remodeled the tumor microenvironment by increasing infiltration of CD4⁺ T cells and a unique natural killer T (NKT) cell subset, highlighting immune reprogramming [90]. Additionally, dual-targeting CAR-NK cells against PD-L1 and MICA/B in lung cancer showed superior cytotoxicity by addressing tumor heterogeneity [91], while in nasopharyngeal carcinoma, combining CAR-NK cells with nivolumab enhanced NK and CD8⁺ T cell activity, resulting in synergistic tumor suppression [92]. Together, these findings underscore the potential of CAR-NK and ICI combination therapies to overcome immune resistance and improve outcomes across diverse solid tumors.

### Oncolytic viruses

Oncolytic viruses (OVs) are capable to selectively infect and replicate within tumor cells, inducing immunogenic cell death that activates both innate and adaptive antitumor immunity. These viruses facilitate the release of tumor-associated antigens (TAAs), damage-associated molecular patterns (DAMPs), and pathogen-associated molecular patterns (PAMPs), which together convert immunologically “cold” tumors into “hot” tumors by enhancing immune infiltration and activation within the tumor microenvironment [93, 94]. This combination of direct oncolysis and immune priming makes OVs a versatile therapeutic platform. Moreover, the efficacy of OVs can be significantly enhanced when combined with immune checkpoint inhibitors such as anti-PD-1 or anti-CTLA-4 antibodies, as evidenced in preclinical and clinical studies. For example, OV infection upregulates PD-L1 expression on tumor cells, sensitizing them to PD-1/PD-L1 blockade and promoting T cell infiltration, thereby reversing immune exclusion [94]. Additionally, genetic engineering enables OVs to deliver immunostimulatory molecules like GM-CSF, IL-12, or checkpoint inhibitor nanobodies directly into the tumor microenvironment, providing localized and sustained immunomodulation [95]. A prime clinical example is Talimogene laherparepvec (T-VEC), a modified HSV-1 encoding GM-CSF, which has demonstrated significantly improved outcomes when combined with checkpoint blockade therapies such as ipilimumab or pembrolizumab in advanced melanoma patients [96].

## Conclusion

Monoclonal antibodies remain a cornerstone for targeting inhibitory immune checkpoints for cancer immunotherapy. However, non-antibody approaches are emerging as promising alternatives. These alternative strategies provide distinct advantages, address specific challenges, and may expand the therapeutic arsenal against cancer.

The clinical outlook for non-antibody ICIs is encouraging, driven by ongoing research and development focused on overcoming current limitations. A growing range of small-molecule inhibitors and peptide-based therapies offer the potential for developing oral medications, while gene-editing technologies provide tailored approaches to modulate patient-specific immune responses and overcome treatment resistance mechanisms.

Furthermore, other innovative approaches—such as LYTACs, PROTACs, and HPMA-based systems—work by degrading immune checkpoint proteins rather than merely inhibiting them, leading to potentially more sustained and robust immune responses.

In parallel, cell- and virus-based platforms such as CAR-NK cells and oncolytic viruses have emerged as synergistic partners for immune checkpoint blockade. These modalities not only provide direct tumoricidal effects but also remodel the tumor microenvironment and enhance immune activation, offering powerful complements or alternatives to monoclonal antibody-based therapies [5, 6].

These strategies seek to enhance therapeutic efficacy while minimizing adverse effects, paving the way for personalized cancer therapies that could significantly improve patient outcomes.

As these non-antibody therapies progress from preclinical studies to clinical application, their integration with current treatment options promises to broaden the scope of immune checkpoint inhibition, ultimately transforming cancer care through more effective and accessible therapeutic interventions.

## Data Availability

No datasets were generated or analyzed during the current study.
